# The Mobile Quarantine Convention

**Published:** 1898-03

**Authors:** 


### EDITORIAL.

The office of THE JOURNAL is 311 and 312 Fitten Building.

Address all communications, and make all remittances payable to THE ATLANTA MEDICAL AND SURGICAL JOURNAL, Atlanta, Ga.

Reprints of articles will be furnished, when desired, at a small cost. Requests for the same should always be made *on the manuscript*.

### THE MOBILE QUARANTINE CONVENTION.

In another place will be found a detailed report of the proceedings of this Convention.

Much good might have come from this Quarantine Convention had not the politicians captured the meeting and turned it into a forum for airing "States' rights" doctrines and completely overriding all scientific discussion relating to quarantine. The method of selecting delegates was in itself faulty, and the charge that the Convention was "packed" with members known to be opposed to national quarantine seemed to have ground when it was found that all the leading essays, with a single exception, were in the hands of advocates of State and municipal quarantine. If this was not convincing, little doubt remained after organization of the Convention was effected. This was purely a "cut and dried" affair. The permanent presiding officer was *presented* to the Convention and took the chair without the formality of a vote, and proceeded to name committees from a prepared list. This at least saved time, although the plan was open to question. In this way the Committee on Resolutions was formed. Great liberality was shown by the chairman in selecting members of this committee, as he did not confine himself to the South Atlantic and Gulf States in gathering proper material, but named "members by invitation" from the States of Illinois, Wisconsin, New York and Maryland. This committee presented a majority report as a memorial to Congress condemning the Caffery bill and recommending the passage of the Spooner bill. The ink in this report was probably well dried before the Convention met. The minority report, signed by two members of the committee, simply urged Congress to adopt a national quarantine system without favoring in its report any of the several quarantine bills now before our national lawmakers. At this juncture the Convention took a recess for dinner, and during the interim a "compromise" resolution was drawn up, and when the Convention was called to order this compromise resolution was presented, and, without discussion or comment, rushed through, the majority and minority reports having been withdrawn. In an another place will be found the "compromise" or substitute resolution in full.

A careful study of this resolution will reveal the fact that the recommendations of the Convention are little, if any, in advance of present national quarantine laws; and few there are who, having had experience during the late epidemic of yellow fever with local quarantine regulations and restriction, will accept with satisfaction the result of the Convention's labor. It may therefore be fairly said of the Mobile Quarantine Convention, that, boiled down, its net result is *nil.* It was as one force, one argument, neutralized by another, with perhaps the balance of credit to the side of the minority.

A good suggestion came near the close of the Convention, and from a gentleman not a delegate, namely: To call a convention of health and quarantine officials of the South Atlantic and Gulf States to meet in Atlanta in April, 1898; and a resolution to this effect was adopted and a committee to arrange the details of the call was appointed. This Convention will deal with the question of uniform rules and regulations of quarantine, and it is hoped that it will also discuss matters pertaining to sanitary affairs in its broadest sense. As invitations are to be extended to all Central and South American countries to participate in the work of the Convention, the proposed meeting assumes international importance, and it may confidently be predicted that some improvement in international quarantine regulations will result.

Hon. C. A. Collier, Mayor of Atlanta and Chairman of the Committee on Calling the Convention, has named April 12, 1898, as the date of assembling, and has issued invitations to that effect.

Atlanta will greet the Convention in its well-known hospitable way.

